# Foot-and-Mouth Disease Virus Capsid Protein VP1 Antagonizes TPL2-Mediated Activation of the IRF3/IFN-β Signaling Pathway to Facilitate the Virus Replication

**DOI:** 10.3389/fimmu.2020.580334

**Published:** 2021-01-08

**Authors:** Junhong Hao, Chaochao Shen, Nannan Wei, Minghao Yan, Xuegang Zhang, Guowei Xu, Dajun Zhang, Jing Hou, Weijun Cao, Ye Jin, Keshan Zhang, Haixue Zheng, Xiangtao Liu

**Affiliations:** State Key Laboratory of Veterinary Etiological Biology, National Foot-and-Mouth Disease Reference Laboratory, Lanzhou Veterinary Research Institute of Chinese Academy of Agriculture Science, Lanzhou, China

**Keywords:** foot-and-mouth disease virus, viral protein 1, tumor progression locus 2, interferon regulatory factor 3/interferon-β, immune escape

## Abstract

Foot-and-mouth disease (FMD) is a severe, highly contagious viral disease of cloven-hoofed animals. In order to establish an infection, the FMD virus (FMDV) needs to counteract host antiviral responses. Tumor progression locus 2 (TPL2), a mitogen-activated protein kinase, can regulate innate and adaptive immunity; however, its exact mechanisms underlying TPL2-mediated regulation of the pathogenesis of FMDV infection remain unknown. In this study, we confirmed that TPL2 could inhibit FMDV replication *in vitro* and *in vivo*. The virus replication increased in *Tpl2*-deficient suckling mice in association with reduced expression of interferon-stimulated genes interferon-α (IFN-α) and myxovirus resistance (MX2) and significantly reduced expression of C-X-C motif chemokine ligand 10 (CXCL10), interferon regulatory factor 3 (IRF3), and IRF7, while the phosphorylation of IRF3 was not detected. Moreover, the interactions between TPL2 and VP1 were also confirmed. The overexpression of TPL2 promoted IRF3-mediated dose-dependent activation of the IFN-β signaling pathway in association with interactions between IRF3 and TPL2. VP1 also inhibited phosphorylation of TPL2 at Thr290, while Thr290 resulted as the key functional site associated with the TPL2-mediated antiviral response. Taken together, this study indicated that FMDV capsid protein VP1 antagonizes TPL2-mediated activation of the IRF3/IFN-β signaling pathway for immune escape and facilitated virus replication.

## Introduction

Foot-and-mouth disease (FMD) is a severe, highly contagious viral disease of livestock worldwide caused by the foot-and-mouth disease virus (FMDV) infection (1). So far, seven serotypes of FMDV have been identified, including O, A, C, SAT1, SAT2, SAT3, and Asia-1. There is no cross-protection between these serotypes, and only a limited cross-reaction is observed between different strains within a single serotype due to extensive sequence variation within the capsid proteins (2). The viral genome, which is about 8.5 Kb, encodes structural proteins including viral protein (VP)1, VP2, VP3, and VP4 and at least 10 nonstructural proteins (including L, 2A, 2B, 2C, 3A, 3B1, 3B2, 3B3, 3C, and 3D) along with several intermediate precursors (1). Many FMDV proteins have evolved with time; these new mutated proteins can promote rapid and efficient replication in host cells *via* antagonism and evade the host immune system (3). For example, the FMDV leader proteinase (Lpro) has a critical role in preventing the synthesis of interferon (IFN)-α/β proteins by reducing IFN-β mRNA and associated interferon-stimulated gene (ISG) products (4). The structural and nonstructural proteins, including VP3 (5, 6), VP1 (7), 3A (8), and 3C^pr^° (3, 9) modulate the innate immune responses *via* distinct mechanisms.

TPL2 is a protein kinase that was initially identified as protooncogene due to its C-terminal truncation product’s role in promoting tumor function (10, 11). TPL2 is ubiquitously expressed and is involved in innate and adaptive immune cells and diverse host tissues, including the liver, lung, and intestines (11–14). The immune-regulatory function of TPL2 can activate the MEK/ERK pathway in response to toll-like receptor (TLR), interleukin-1 receptor (IL-1R), or tumor necrosis factor receptor signaling (15, 16). While TPL2 is required for IFN-α production in plasmacytoid dendritic cells (pDCs) and to promote IFN-γ secretion by CD4+ T cells, it is also a potent negative regulator of IFN-β in macrophages and DCs (17, 18). Consequently, TPL2 is essential for mounting effective immune responses during infections. *Tpl2* ^-/-^ mice have shown to be susceptible to infection with *Toxoplasma gondii* (18), *Listeria monocytogenes* (19), *Mycobacterium tuberculosis* (20), and *B streptococcus* (21). Surprisingly, information on TPL2-mediated contributions to host defense against viruses are still rather limited and contradictory. Although it has been identified as a major regulator of type I and type II IFNs, the mechanisms underlying TPL2-mediated regulation of type I IFNs have not yet been examined in detail. The main effectors that both initiate and amplify host cell antiviral responses are a family of transcription factors known as interferon regulatory factors (IRFs) (22). IRFs have been implicated in numerous cellular activities. For example, IRF1, IRF2, IRF3, IRF5, IRF7, and IRF9 have been directly implicated in one or more aspects of the antiviral response (23–30). IRF3 and IRF7 are directly involved in the transcriptional induction of IFNs and also have the capacity to induce ISGs in an IFN-I-independent manner (31–34). Moreover, IRF3, which serves as the key signal mediator and transcription factor for the induction of IFN-I, undergoes phosphorylation followed by homodimer formation and nuclear translocation to initiate gene transcription in response to pathogen infection. Understanding the link between phosphorylation and activation of IRF3 has been a topic of interest with respect to innate immunity. However, it remains unclear in which host proteins interact with FMDV VP1 and regulate FMDV replication.

This study found that TPL2 can inhibit FMDV replication both *in vitro* and *in vivo*; FMDV VP1 interacts with TPL2 and inhibits its phosphorylation at Thr290. Also, TPL2 promotes the activation of the IRF3/IFN-β signaling pathway, while VP1 inhibits TPL2-mediated IFN-β, as well as expression of mRNAs that encode several of the ISGs. Taken together, our results revealed that the interactions between VP1 and TPL2 result in inhibition of IFN-β-mediated signaling and enhance virus replication.

## Material and Methods

### Materials

Porcine kidney (PK-15) cells and cells of the human embryonic kidney cell line (HEK293T) were cultured in Dulbecco’s modified Eagle’s medium (Gibco) supplemented with 10% fetal bovine serum (Gibco) in a humidified atmosphere containing 5%CO_2_/95% air at 37°C. Virus infection experiments were carried out as previously described (35). Foot-and-mouth disease strain A/GDMM/CHA/2013 and Sendai virus (SeV) are maintained by State Key Laboratory.

Rabbit anti-IRF3 and anti-phospho-IRF3 antibodies were purchased from Cell Signaling Technology (Danvers, MA, USA). RIG-I, TBK1, p-TBK1, IKK- α were acquired from Sigma-Aldrich. Alexa fluor-conjugated secondary antibodies for indirect immunofluorescence (IFA) were purchased from Life Technologies. Rabbit polyclonal anti-TPL2 and rabbit polyclonal anti-TPL2-T290 antibodies were obtained from Abcam. Mouse monoclonal anti-c-Myc and mouse monoclonal anti-c-Flag were purchased from Sigma Aldrich, and mouse anti-β-actin was bought from Thermo Fisher Scientific.

### Construction of Eukaryotic Expression Plasmids and Transfection

TPL2 primers were designed based on the TPL2 gene sequence listed in GenBank (NM007746.2) and used to facilitate the cloning of relevant sequences into the pCMV3N-Myc vector. Flag-VP1 and the expression vector pCAGGS were constructed and provided by our lab. The IFN-β promoter-luciferase reporter plasmids, pRL-TK plasmid, and various HA-tagged plasmids used in this study were a kind gift from Professor Hongbing Shu (Wuhan University, China) (36). All newly-constructed plasmids were sequenced, and the correct insertion of each gene was verified. The DNA plasmids were transfected into the cells using the transfection reagent Lipofectamine™ 3000 (Invitrogen) according to the manufacturer’s instructions.

### TPL2 Gene-Deleted PK-15 Cell Line Established Using CRISPR/Cas9

To construct the TPL2 gene-deleted (TPL2-KO)-PK-15 cell line, two specific CRISPR-Cas9 sgRNAs were designed to target the second exon of porcine-derived TPL2. The sgRNA was cloned into the lentiGuide-EGFP vector using the BsmBI terminal sequence to construct the lentiGuide-EGFP-TPL2-sg RNA Lentiviral Expression Plasmid. The lentiGuide-EGFP-TPL2-sg RNA lentiviral expression plasmid and virus packaging helper plasmid were then co-transfected into PK-15 cells in order to target TPL2 for gene knockout; Lenti-Guide-EGFP vector and virus packaging helper plasmid were co-transfected into PK-15 cells as a control. Forty-eight hours after transfection, the expression of the fluorescent protein was observed under a fluorescence microscope, indicating that the plasmid was successfully transfected into PK-15 cells. The virus was concentrated and used to infect cells; positive clones were identified by green fluorescence using flow cytometry.

### PK-15 Cell Line Stably Expressing Porcine TPL2

The recombinant lentiviral vector Lv-TPL2 was constructed by chemical synthesis of the porcine TPL2 gene as per datafile XM_021064737.1 at the National Center for Biotechnology Information, Bethesda, MD, USA. The two restriction sites used were *Xba* I and *Bam*H I. The appropriate restriction enzyme sites were ligated into the multiple cloning site of the lv-pCDH vector. Restriction sites for Xba I and *Bam*H I were introduced at the 5′and 3′ends of the target DNA fragment. Recombinant lentiviral vector Lv-MAP3K8 was also obtained.

### Luciferase Activity

HEK293T cells (1 × 10^5^) were cultured in 24-well plates, and the monolayer cells were transfected with 0.2 μg of Myc vector, Myc-TPL2, HA-IRF3-expressing plasmids, and 0.1 μg of IFN-β-promoter-driven luciferase reporter plasmid together with 0.02 μg of internal control pRL-TK reporter plasmid. The transfected cells were then mock-infected or infected with SeV at 24 h post-transfection (hpt). The luciferase activities were measured using a dual-luciferase reporter assay kit (Promega) according to the manufacturer’s protocol.

To evaluate adaptor molecule-induced IFN-β-promoter activation assay, HEK293T cells (1 × 10^5^) were seeded on 24-well and transfected with 0.1 μg of the IFN-β, ISRE-promoter-driven luciferase reporter plasmids, 0.01 μg pRL-TK, 0.1 μg adaptor plasmids, and Myc-TPL2 plasmids. The dual-luciferase activity was measured at 24 hpt. All experiments were performed in triplicate. The results are presented with SEM (standard error of the mean) from three independent experiments.

### Real-Time qPCR (qRT-PCR)

Total RNAs were extracted using TRIzol Reagent (Invitrogen, USA), and cDNA was synthesized from the extracted RNA samples. The SYBR Premix Ex Taq reagents (Takara) were used to quantify RNA copy numbers. The generated cDNA was used as a template to detect the expression of FMDV RNA and host cellular mRNA. The gene encoding glyceraldehyde-3-phosphate dehydrogenase (GAPDH) gene was used as an internal control. Relative expression of mRNA was calculated using the comparative cycle threshold (2^−ΔΔCt^) method. All the experiments were repeated three times. The primers used to amplify the genes are listed in **Table 1**.

**Table 1:** Primers used in this study.

Gene	Sequences(5’-3’)
TPL2	F: TCGACCAAAGCCGACATCTA
	R: TATCAGCTCCCTCATGCCTG
GAPDH	F: ACATGGCCTCCAAGGAGTAAGA
	R: GATCGAGTTGGGGCTGTGACT
FMDV	F: CACTGGTGACAGGCTAAGG
	R: CCCTTCTCAGATTCCGAGT
M-GAPDH	F: CATGTTCCAGTATGACTCCACT
	R: GTAGACTCCACGACATACTCAG
M-TPL2	F: AACCTTTATGCAAGTGAAGAGCC
	R: TCCACGGTCCCATATCTGACA
M-TPL2-KO	F: CAGTTTAAGCCATCGGATGTGG
	R: AGGACGGCACCATATAACTCA
M-IFN-β	F: AGCTCCAAGAAAGGACGAACA
	R: GCCCTGTAGGTGAGGTTGAT
M-CXCL10	F: CCAAGTGCTGCCGTCATTTTC
	R: GGCTCGCAGGGATGATTTCAA
M-ISG15	F: GTGATGCTAGTGGTACAGAACT
	R: TCTTAAGCGTGTCTACAGTCTG
M-IRF3	F: CTGACAATAGCAAGGACCCTTA
	R: AGGCCATCAAATAACTTCGGTA
M-IRF7	F: AAATAGGGAAGAAGTGAGCCTC
	R: CCCTTGTACATGATGGTCACAT
ISG56	F: TCATCAGGTCAAGGATAGTC
	R: CCACACTGTATTTGGTGTCTAGG
IFN-β	F: GGCTGGAATGAAACCGTCAT
	R: TCCAGGATTGTCTCCAGGTCA
H-MXA	F: ACCTCGTGTTCCAACTGAAG
	R: GTGTGATGAGCTCGCTGGTA
H-GBP1	F: CGAGGGTCTGGGAGATGTAG
	R: TAGCCTGCTGGTTGATGGTT
H-ISG20	F: CTCCTGCACAAGAGCATCCA
	R: CGTTGCCCTCGCATCTTC
H-IFN-β	F: TCTTTCCATGAGCTACAACTTGCT
	R: GCAGTATTCAAGCCTCCCATTC
H-OXAS1	F: TCCACAGCCTCACTTCATTCC
	R: ACATTAGACATTACCCTCCCATCAG
H-ISG56	F: GCCTTGCTGAAGTGTGGAGGAA
	R: ATCCAGGCGATAGGCAGAGATC

### Indirect Immunofluorescence Assay (IFA)

HEK293T cells were grown on glass-bottom dishes, cultured overnight, and then transfected with 2 μg of HA vector or HA-IRF3 and 2 μg of Myc vector or Myc-TPL2 plasmids for 36 h. Cells were mock-infected or infected with SeV at 24 hpt and cultured for 16 h. The cells were then fixed with 4% paraformaldehyde for 30 min and permeabilized with 0.1% Triton X-100 for 15 min. Fixed cells were incubated in 5% bovine serum albumin at 4°C for 4 h, followed by incubation with the appropriate primary antibody, and then with Alexa Fluor 488-or 594-conjugated secondary antibody. The images were acquired with a laser-scanning confocal microscope (Leica SP8, Solms, Germany).

### Co-Immunoprecipitation and Western Blotting

Co-immunoprecipitation (Co-IP) assays were performed as previously described (37). Briefly, HEK293T cells were cultured in 10 cm^2^ dishes, and the monolayer cells were transfected with various plasmids as indicated. For each sample, 0.5 ml of cell lysate was incubated with 0.5 mg of specific antibody or control IgG and 40 μl of protein G-Sepharose in 20% ethanol (GE Healthcare) 12 h. The protein-G-Sepharose beads were washed 3 times with 1 ml of lysis buffer containing 500 mM NaCl. The precipitates were analyzed by immunoblotting.

For Western blotting (WB), target proteins were separated by sodium dodecyl sulfate-polyacrylamide gel electrophoresis (SDS-PAGE) and transferred onto an immobilon-p membrane (Millipore, USA). Non-specific binding sites were blocked, and the membrane was then incubated with appropriate primary and secondary antibodies. Antibody-antigen complexes were visualized using enhanced chemiluminescence detection reagents (Thermo, USA) (38).

### Construction of *Tpl2* Gene-Knockout Suckling Mice and Challenge With FMDV

DNA was extracted from the mouse tail using a DNA extraction kit to identify C57BL/6 mouse genes. Minced tissue was placed in a 2 ml centrifuge tube with 300 ul lysis buffer with 1 mM phenylmethylsulfonyl fluoride (PMSF). The tissue was homogenized 3 times for 30 s each. Then, 100–200 μl of lysis buffer was added to the tissue sample, which was then mixed on ice or lysed at 4°C for 10 min. The lysate was centrifuged at 12,000 rpm for 15 min, and the supernatant was collected. Consequently, 5X SDS loading buffer was added, and the mixture was centrifuged after boiling for 10 min.

For analysis of the antiviral responses to FMDV infection and replication, wild type C57BL/6 suckling mice (5 in each group) were intravenously injected with FMDV (7×10^4^ plaque-forming units (pfu)). FMDV-associated expression of mRNAs encoding IFN-α, MX2, CXCL10, and IRF3, IRF7 were analyzed by qRT-PCR. The survival of the mice was monitored as indicated (39, 40).

All animal studies (including the mice euthanasia procedure) were done in compliance with the regulations and guidelines of Lanzhou Veterinary Research Institute (Chinese Academy of Agriculture Science) institutional animal care and conducted according to the AAALAC and the IACUC guidelines (License No. SYXK [GAN] 2014–003).

### Statistical Analysis

All data were present as mean values ± standard error (mean ± SE) of three independent experiments. Two-tailed Student’s t-tests were used to analyze the significance of the data. Differences were considered to be statistically significant at *P<0.05, and highly significant at **P<0.01 and ***P < 0.001.

## Results

### TPL2 Inhibits FMDV Replication *In Vitro* and *In Vivo*

**Figure 1:**
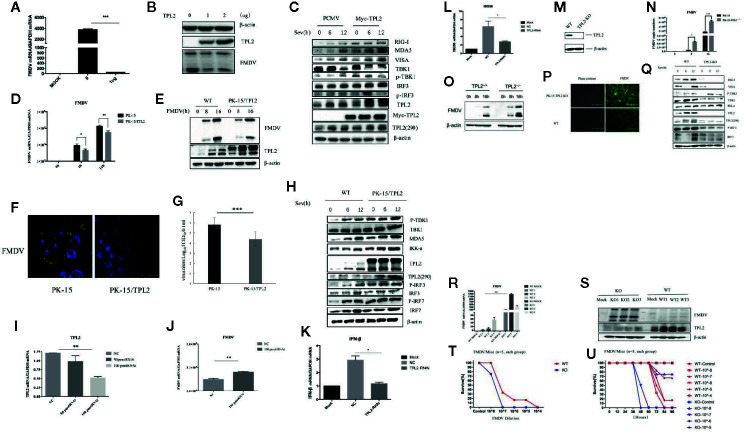
Host TPL2 inhibits the replication of FMD virus (FMDV). **(A, B)** The PK-15 cell line was transfected with 0 μg and 1 μg of Myc-TPL2 plasmid. Twenty-four hours after transfection, cells were infected with FMDV (MOI = 0.5). Virus titer was evaluated 12 h later using real-time qPCR (qRT-PCR) and Western blotting (WB). **(C)** HEK293T cells were transfected with 5 μg each of the plasmids. Twenty-four hours after transfection, the cells were infected with SeV and then evaluated after 0, 6, and 12 h. WB was performed to detect immunoreactive RIG-I, MDA5, VISA, TBK1, P-TBK1, IRF3, IRF7, and phosphorylated at Thr290 [TPL2 (290)]. **(D, E)** The PK-15/TPL2 cell line that stably expresses TPL2 and WT PK-15 cells were collected at 0, 8, and 16 h after infection with FMDV (MOI = 0.5). FMDV replication was evaluated by qRT-PCR and WB. **(F)** PK-15/TPL2 and WT PK-15 cells were infected with FMDV and analyzed after 16 h using IFA. **(G)** PK-15/TPL2 and WT PK-15 cells were infected with FMDV (MOI = 0.5); viral titers were determined using the 50% tissue culture infective dose (TCID_50_) assay. **(H)** PK-15/TPL2 and WT PK-15 cells line were collected at 0, 6, and 12 h after SeV infection for detection of immunoreactive RIG-I, MDA5, VISA, TBK1, P-TBK1, IRF3, IRF7, TPL2 (290) by WB. **(I)** Effect of TPL2-RNAi plasmid on the expression of the endogenous TPL2 gene. **(J)** TPL2-RNAi plasmid was transfected into PK-15 cells. After 24 h, the transfected cells were infected with FMDV (MOI = 0.5); qRT-PCR was performed to evaluate FMDV-associated gene transcription. **(K, L)** WT or TPL2-RNAi-transfected HEK293T cells were infected with SeV; 12 h later, transcription of genes encoding IFN-β and ISG56 was evaluated by qRT-PCR. **(M)** WB evaluated the TPL2 protein level in TPL2 gene-deleted PK-15 cells. **(N, O)** TPL2 gene-deleted PK-15 cells were infected with FMDV (MOI = 0.5). Cells were collected 0, 8, and 16 h later to evaluate virus titer and FMDV-associated gene transcription by qRT-PCR. **(P)** WT and TPL2 gene-deleted PK-15 cells were infected with FMDV (MOI = 0.5); after 16 h, cells were evaluated by immunofluorescence (IFA). **(Q)** WT PK-15 and TPL2 gene-deleted PK-15 cells were mock-infected or infected with SeV and collected at 0, 6, and 12 h to evaluate phosphorylation of immunoreactive RIG-I, MDA5, VISA, TBK1, P-TBK1, IRF3, IRF7, and TPL2 (290) by WB. **(R, S)** FMDV replication was evaluated in WT and *Tpl2^-^* ^/-^ suckling mice by qRT-PCR and WB. **(T, U)** Survival of WT and *Tpl2^-^* ^/-^ suckling mice was evaluated after intramuscular injection of 7×104 pfu of FMDV.

We first examined whether TPL2 is involved in the regulation of virus-triggered IFN-β signaling. An overexpression of TPL2 resulted in significant inhibition of FMDV replication at the levels of gene transcription and protein levels in porcine PK-15 cells (**Figures 1A, B**). As shown in **Figure 1C**, SeV-triggered activation of the IFN-β pathway was markedly higher in the TPL2-overexpressing 293T cells in comparison to controls. FMDV replication was significantly inhibited in the stable PK-15/TPL2-overexpressing cell line, as confirmed by IFA, WB, and qRT-PCR (**Figures 1D–F**). We also observed a similar trend in PK-15 and PK-15/TPL2 cells (**Figure 1G**). As shown in **Figure 1H**, SeV-triggered phosphorylation of TBK1 and IRF3 was markedly higher in PK-15/TPL2 cells when compared with controls. Taken together, these results suggested that TPL2 potentiates virus-triggered activation of the IFN-β signaling pathway and inhibits FMDV replication.

Further, we investigated the function of endogenous TPL2 with respect to SeV-triggered production of type I IFNs. RNAi plasmids targeting TPL2 were constructed. Transfection with TPL2-RNAi plasmids significantly reduced the expression of endogenous TPL2 and promoted FMDV replication in PK-15 cells (**Figures 1I–J**). In addition, SeV-triggered transcription of IFN-β and ISG56 genes was markedly reduced in the TPL2-knockdown 293T cells compared with that observed in control cells (**Figures 1K, L**). Collectively, these results suggested that TPL2 knockdown results in diminished levels of virus-triggered induction of IFN-β.

In order to examine the roles of TPL2 in the antiviral innate immune response, a TPL2 gene-knockout PK-15 cell line was established using the CRISPR/Cas9 method. Reduced levels of TPL2 protein were observed in TPL2-knockout PK-15 cells by WB (**Figure 1M**). Furthermore, we found that FMDV replication was markedly increased in TPL2-knockout PK-15 cells compared with wild-type cells (**Figures 1N–P**). Further experiments indicated that SeV-triggered phosphorylation of TBK1 and IRF3 was markedly reduced in TPL2-knockdown cells compared to control cells (**Figure 1Q**).

Next, we explored the role of TPL2 as a mean of host defense against FMDV infection *in vivo*. After *Tpl2* gene-deleted (*Tpl2* ^-/-^) suckling mice were infected with FMDV virus, higher levels of FMDV replication were detected in the gene-deleted strain when compared with the wild-type mice (WT; **Figures 1R, S**). Suckling *Tpl2* ^-/-^ mice died in response to FMDV, while WT suckling mice survived with the same dose (*P* < 0.05; **Figures 1T, U**). Collectively, these results suggested that TPL2 has a critical role in mediating IFN-β signaling and FMDV replication.

**Figure 2:**
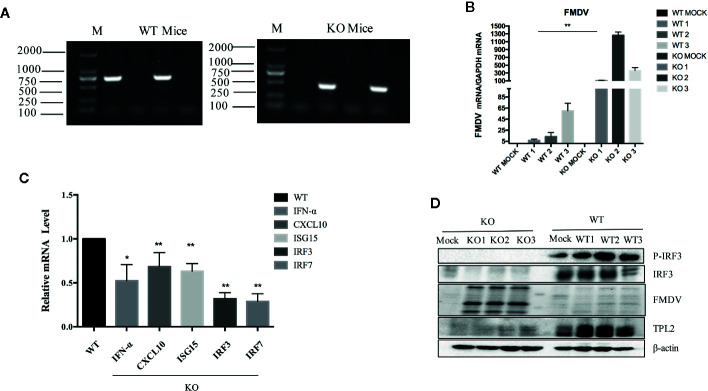
TPL2 is essential to inhibit FMD virus (FMDV) replication *in vivo*. **(A)** Primers targeting TPL2 knockout region. DNA from WT and *Tpl2^-^* ^/-^ suckling mice was extracted. After the PCR procedure, the expected size of PCR fragments appeared in the WT and *Tpl2^-^* ^/-^ group. **(B)** WT and *Tpl2^-^* ^/-^ suckling mice were used to evaluate FMDV-associated gene transcription by real-time qPCR (qRT-PCR). **(C)** Total RNA was extracted from the carcasses of WT and *Tpl2^-^* ^/-^ suckling mice to evaluate transcription of genes encoding IFN-α, MX2 and CXCL10, IRF3, and IRF7 by qRT-PCR. **(D)** Protein in lysates extracted from carcasses of WT and *Tpl2^-^* ^/-^ suckling mice were used to evaluate phosphorylation of IRF3 and infection with FMDV(n = 5 per group).

Suckling *Tpl2* ^-/-^ mice were successfully knocked out (**Figure 2A**). The impact of this gene-deletion on FMDV was verified by qRT-PCR analysis (**Figure 2B**). This was associated with a complete block of the phosphorylation of IRF3 (**Figure 2D**). IRF3 and IRF7 are transcription factors required for the antiviral response, not only for the induction of IFN-I but also for establishing an antiviral state. Moreover, we observed significant reductions in mRNAs expression encoding IFN-α, MX2, CXCL10, IRF3, and IRF7 in the tissues of FMDV-infected *Tpl2*-gene-deleted suckling mice compared with WT littermates (**Figure 2C**). These findings suggested that TPL2-mediated phosphorylation of IRF3/IFN-β is a critical event associated with positive regulation of antiviral immunity.

### FMDV VP1 Interacted With TPL2

**Figure 3:**
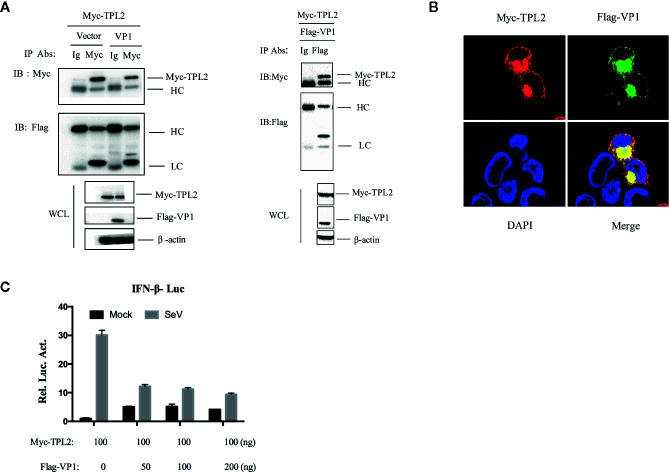
VP1 interacted with TPL2. **(A)** HEK293T cells were transfected with plasmids expressing FMDV VP1 (8 μg) and TPL2 (8 μg); cell lysates were incubated with anti-Myc and control IgG antibodies. Co-immunoprecipitation complexes were detected with anti-Flag and anti-Myc. **(B)** HEK293T cells were transfected with plasmids expressing Myc-TPL2 (1 μg) and Flag-VP1 (1 μg) and subjected to immunohistochemical staining; as shown Myc-TPL2 (red), Flag-VP1 (green), and cellular nuclei were stained blue with DAPI. **(C)** HEK293T cells were transfected with expression plasmids as indicated and then mock-infected or infected with SeV. After 12 h, luciferase assays were performed.

For further evaluation of the interaction between TPL2 and VP1, a Co-IP assay was performed that included co-transfection of HEK293T cells with plasmids expressing Flag-VP1 and Myc-TPL2 plasmids. After immunoprecipitation with either anti-Flag or anti-Myc antibody, we detected Flag-VP1 co-precipitated with Myc-TPL2 (**Figure 3A**). Co-localization of VP1 and TPL2 was examined by double-label immunofluorescence and confocal microscopy. The results showed a clear co-localization of VP1 and TPL2 (**Figure 3B**). Furthermore, the overexpression of VP1 led to decreased levels of IFN-β through a dose-dependent mechanism associated with TPL2 (**Figure 3C**). To sum up, these data indicated that the FMDV VP1 interacted specifically with host TPL2.

### TPL2 Activate IRF3-Mediated IFN-β Signaling Pathway

**Figure 4:**
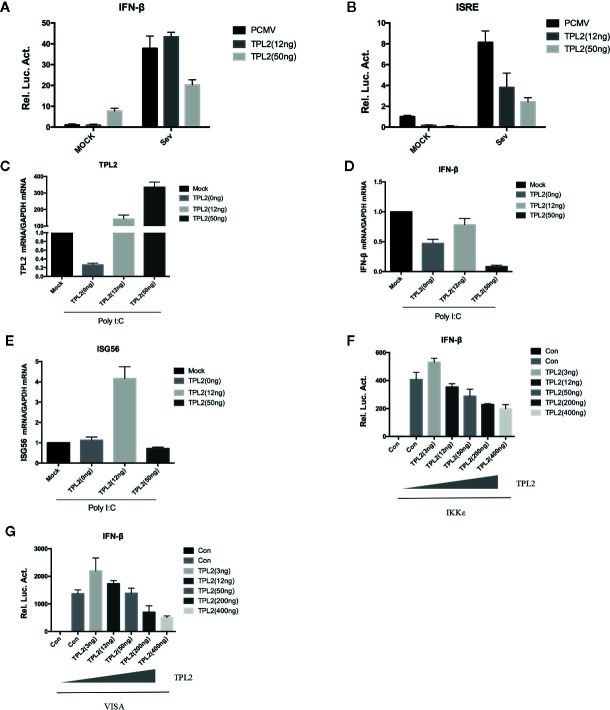
TPL2 activates the IFN-β signaling pathway. **(A, B)** HEK293T cells were transfected with 3, 12, or 50 ng of TPL2 empty-loaded PCMV and TPL2 plasmids and then infected with SeV. Cells were collected 12 h later for dual-luciferase assays. **(C–E)** HEK293T cells were transfected with 12 or 50 ng of TPL2 unloaded PCMV and TPL2 plasmids. After 24 h, transfected cells were stimulated with poly (I:C) at 1 μg/ml for an additional 12 h. **(F, G)** HEK293T cells were transfected with exogenous mitochondrial antiviral signaling protein (MAVS) and IKKϵ and evaluated with respect to dose-dependent expression of TPL2.

In reporter assays, SeV -triggered activation of the IFN-β promoter and the interferon-specific response elements (ISREs) were markedly higher in cells that were overexpressing TPL2. It was observed that TPL2-mediated dose-dependent inhibition at the IFN-β promoter and ISREs (**Figures 4A, B**). qRT-PCR indicated marked inhibition of poly(I:C)-triggered transcription of the IFN-β and ISG56 in response to overexpression of TPL2 when compared with control cells (**Figures 4C–E**). Pattern recognition receptor activation resulted in the assembly of a signaling complex at the mitochondrial membrane that included virus-induced signaling adaptor (VISA) and engagement of both the classical IKK and IKK-related kinases, as well as mitogen-activated protein kinases (MAPKs), culminating in the activation of NF-κB, IRF, and AP1, respectively. Both VISA and IKK promoted activation of IFN-β promoter and ISRE; these responses are decreased in a dose-dependent manner in response to TPL2 (**Figures 4F–G**). Taken together, these results indicated that TPL2 mediates a dose-dependent inhibition virus-induced activation of the IFN-β signaling pathway.

**Figure 5:**
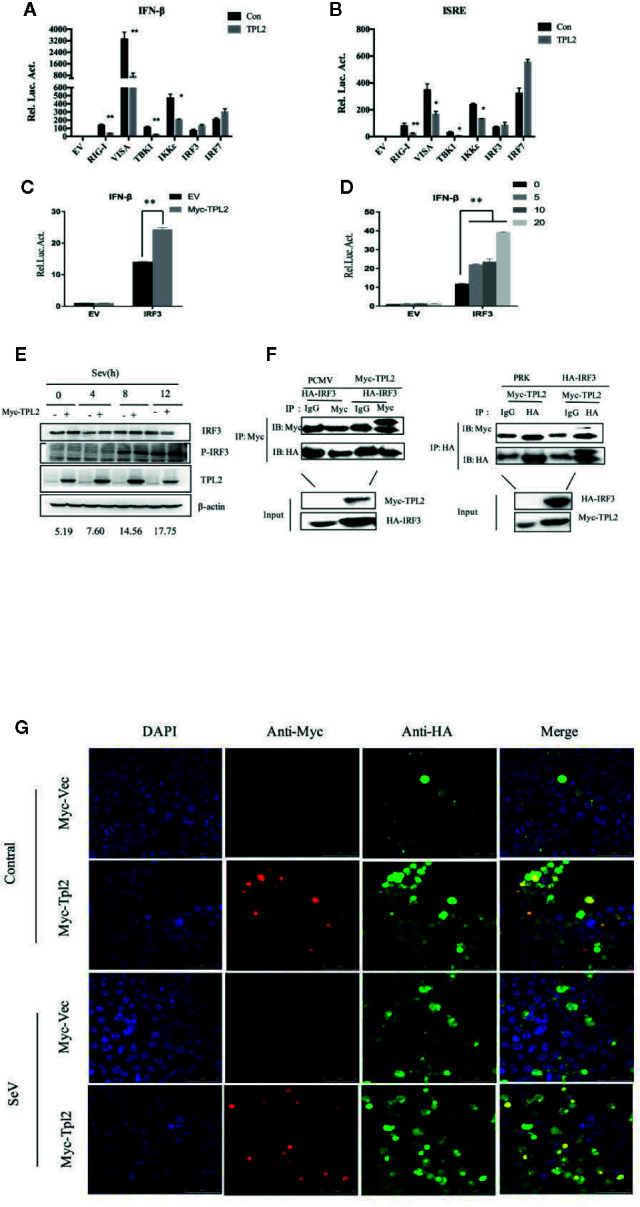
TPL2 regulates virus-triggered signaling at the level of IRF3. **(A, B)** HEK293T cells were transfected with 100 ng each of ISRE and promoter IFN-β reporter plasmids and 100 ng each of the TPL2 expression plasmid together with expression plasmids for RIG-I, VISA, TBK1 IKKϵ, and IRF3. After 24 h, reporter analysis was performed. **(C, D)** Cells were transfected with 0, 5, 10, or 20 ng of the Myc-TPL2 expression plasmid. Cells were collected 24 h later and analyzed using a dual-luciferase reporter assay. **(E)** Cells were transfected with TPL2/pCMV3N-Myc (1 μg) and were infected with SeV for 24 h and collected at 0, 4, 8, 12 h after infection. The results were compared with no-transfection controls. **(F)** HEK293T cells were transfected with expression plasmids encoding HA-IRF3 and Myc-TPL2 (5 μg each). **(G)** HEK293T cells were transfected with expression plasmids encoding HA-IRF3 and Flag-TPL2 (0.5 μg each). After 24 h, cells were left untreated or infected with SeV. Immunofluorescence experiments were performed 12 h later.

TPL2 inhibited the activation of the IFN-β promoter and ISREs mediated by RIG-I, VISA, TBK1, and IKKϵ, but not IRF3 (**Figures 5A, B**). These results suggested that TPL2 targets either IRF3 or a signaling step upstream. To determine whether TPL2 has an impact on IRF3, we performed reporter assays. The results of these assays revealed that the overexpression of IRF3 resulted in the production of IFN-β. Likewise, the overexpression of TPL2 promoted a dose-dependent activation of the IRF3-induced IFN-β signaling pathway (**Figures 5C, D**). Moreover, WB revealed that TPL2 promoted the phosphorylation of IRF3. This result also indicated TPL2 promoted activation of IRF3/IFN-β signaling by enhancing IRF3 phosphorylation (**Figure 5E**).

Since TPL2 targets IRF3 or a signaling step upstream of IRF3, we evaluated the potential for interactions between TPL2 and IRF3. We identified a direct interaction between TPL2 and IRF3 (**Figure 5F**). In addition, confocal microscopy experiments revealed that TPL2 co-localized with IRF3 in the cell nucleus in response to SeV infection (**Figure 5G**). These results suggested that TPL2-mediated interactions with IRF3 may promote IRF3 translocation into the nucleus.

### VP1-Mediated Interactions With TPL2 (Thr290) to Inhibit IRF3/IFN-β Signaling

**Figure 6:**
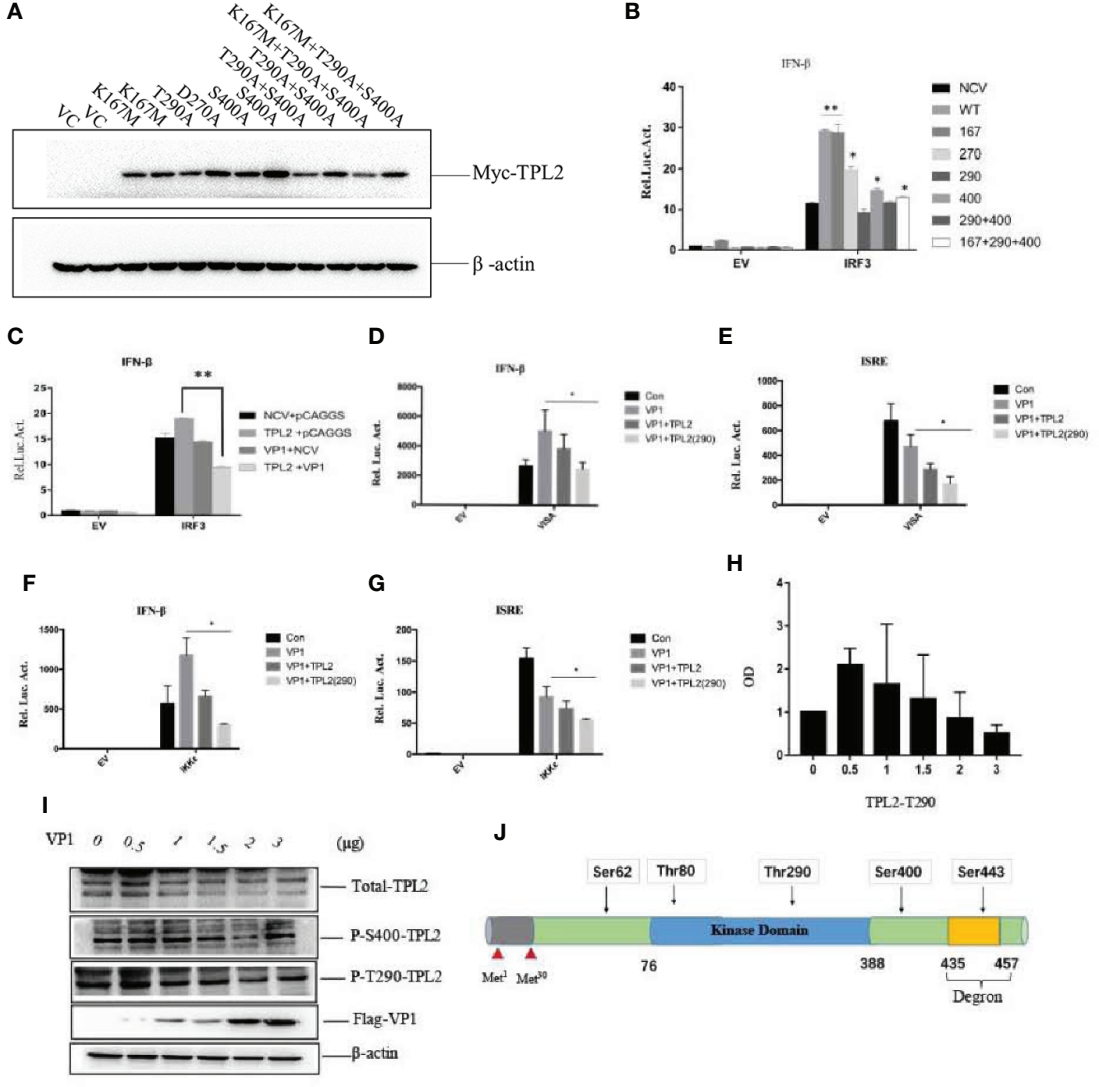
FMDV VP1 inhibits TPL2 phosphorylation at the Thr290 site. **(A)** HEK293T cells were transfected with TPL2 functional site mutant plasmids. Vector plasmids (pCMV-3N-Myc) were used as vector control (VC). Except for plasmids of T290A and D270A, the others occupied two lanes. **(B)** HEK293T cells were transfected with 10 ng WT Myc-TPL2, 10 ng mutant Myc-TPL2, 100 ng IFN-β-Luc reporter plasmid, 10 ng PRL-TK internal reference plasmid, the pCMV−3N-Myc empty vector, and the HA-IRF3 plasmid. Cells were collected at 24 h after transfection for evaluation by dual-luciferase assay. **(C)** HEK293T cells were transfected separately with Myc-TPL2 (10 ng), Flag-VP1 (100 ng) with 100 ng IFN-β-Luc reporter plasmid, 10 ng PRL-TK internal reference plasmid, pCMV-3N-Myc empty vector, pCAGGS empty vector, and HA-IRF3 plasmid. 24 h later, activity was detected using the dual-luciferase assay. **(D–G)** HEK293T cells were transfected separately with IKKϵ, VISA, Myc-TPL2, TPL2(290) at 10 ng, and Flag-VP1 together with 100 ng of an IFN-β-Luc reporter plasmid, 10 ng PRL-TK internal reference plasmid, pCMV-3N-Myc empty vector, pCAGGS empty vector and HA-IRF3 plasmid. **(H, I)** Cells were transfected with 0, 0.5, 1, 1.5, 2, or 3 μg of VP1 expression plasmid together with the pCAGGS empty vector; cells were then collected at 24 h after transfection to evaluate protein expression and gene transcription. **(J)** Sketch map of TPL2 structure and phosphorylation site.

A series of TPL2 mutants were constructed. Thr290 and Ser400 were converted to alanine (Ala), and Lys167 was mutated to methionine (Met) (**Figure 6J**). Plasmids expressing TPL2 with mutations at key functional sites were then transfected into HEK293T cells (**Figure 6A**). Briefly, the TPL2 T290A mutant protein did not promote IRF3-mediated activation of IFN-β signaling as determined by reporter assays (**Figure 6B**).

Next, we explored whether the FMDV VP1 had an impact on TPL2- mediated IRF3-induced IFN-β activation. The results revealed that VP1 inhibited TPL2 mediated IRF3 activation of the IFN-β signaling pathway (**Figure 6C**). Additionally, the levels of secreted IFN-β induced by the actions of VISA and IKK were inhibited by TPL2 (Thr290) in comparison with levels detected in control cells (**Figures 6D–G**). WB confirmed that the expression of total TPL2 protein as well as the Ser400 phosphorylated form, did not significantly alter in response to increasing levels of VP1. Phosphorylation at Thr290 significantly decreased with increasing levels of VP1 transfection (**Figures 6H, I**). Collectively, these results suggested that the Thr290 site of TPL2 was important for IFN-I signaling activation.

**Figure 7:**
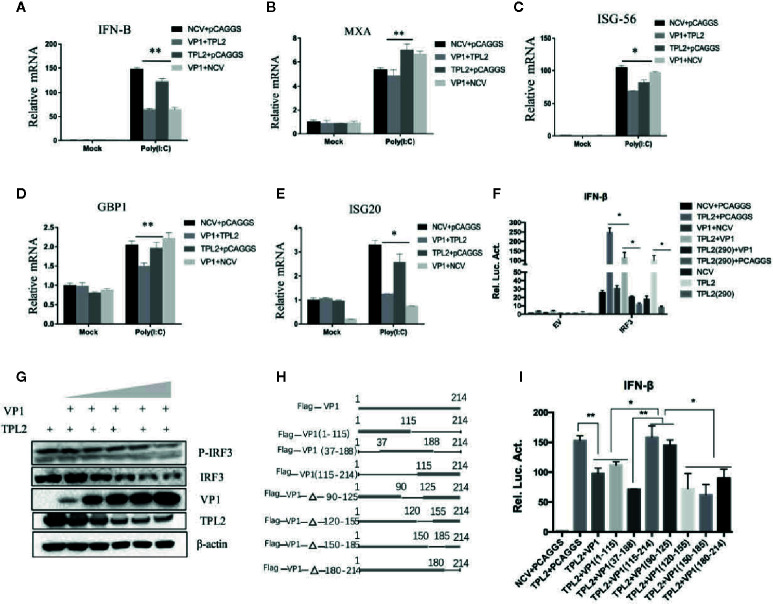
VP1-mediated TPL2 inhibition of the IFN-β signaling pathway. **(A–E)** HEK293T cells were transfected with expression plasmids encoding Flag-VP1, and Myc-TPL2 followed dual-luciferase assay. Quantitative RT-PCR was used to evaluate the expression of genes encoding IFN-β, MXA, ISG-56, GBP1, and ISG-20. **(F)** HEK293T cells were transfected with expression plasmids encoding Flag-VP1, Myc-TPL2, TPL2 (290), and IRF3 and detected using the dual-luciferase reporting system. Each dataset is the result of three or more independent experiments. **(G)** HEK293T cells were transfected with 0, 0.5, 1, 2, 4, or 8 μg of the Flag-VP1 expression plasmid with 0.5 μg TPL2 expression plasmid. After 24 h, cells were collected, and the lysate was probed with anti-IRF3, anti-P-IRF3, anti-Myc, and anti-Flag-VP1 by Western blotting (WB). **(H)** Schematic diagram of VP1 truncated mutants. **(I)** HEK293T cells were transfected with plasmids encoding Myc or Myc-TPL2 and plasmids expressing Flag-VP1 or Flag-VP1-truncation products. 24 h later, IFN-β was measured by luciferase assay.

The qRT-PCR indicated that poly(I:C)-triggered transcription of the genes encoding IFN-β, MXA, GBP1, ISG20, and ISG56 was markedly inhibited in association with overexpression of TPL2 and VP1 in comparison with control cells (**Figures 7A–E**). In the reporter assays, both TPL2 and IRF3 overexpression promoted IRF3-induced activation of the IFN-β signaling pathway. In the presence of the TPL2 with T290A mutation, VP1 was capable of significant inhibition of IFN-β activation (**Figure 7F**). WB confirmed that the overexpression of VP1 inhibits the interaction of TPL2 and IRF3 total protein, thereby decreasing the phosphorylation of IRF3 (**Figure 7G**). To identify the region or regions of FMDV VP1 that were responsible for this interaction, we generated a series of Flag-tagged truncated VP1 constructs (**Figure 7H**). Our results revealed that the N-terminal 115–214 amino acids of VP1 and the deletion mutant (VP1-△90–125) were not capable of interfering with IFN-β signaling activation. These results suggested no functional sites within the amino-terminal 115–214 region of VP1, although there was at least one functional site or region located at the sequence within the N-terminal 90–115 amino acids of VP1 (**Figure 7I**).

**Figure 8:**
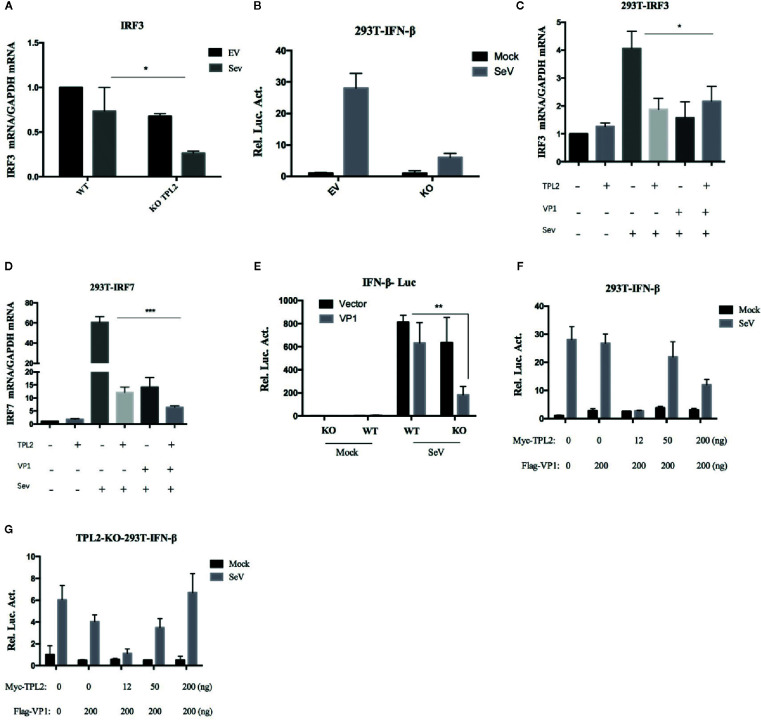
overexpression of TPL2 restored VP1-induced inhibition of IFN-β signaling. Detection of IRF3 and IFN-β transcription in HEK293T-WT and HEK293T-*TPL2^-/-^* cells both with or without SeV infection using RT-PCR **(A)** and luciferase assays **(B)**. **(C, D)** HEK293T cells were transfected with Flag-VP1 or TPL2. At 24 hpt, the cells were mock-infected or infected with SeV for an additional 12 h; qRT-PCR was used to detect IRF3 and IRF7. **(E)** HEK293T-WT and HEK293T-*TPL2* ^-/-^ cells were transfected with Flag-VP1, empty vector PCAGG, and Flag-VP1 or TPL2 expression plasmids **(F, G)**. At 24 hpt, the cells were mock-infected or infected with SeV for an additional 12 h after which luciferase assays were performed.

HEK293T-*TPL2* ^-/-^ cell line was constructed to determine the mechanisms underlying the impact of TPL2 on the VP1-mediated inhibition of the IFN-β response. SeV infection of these cells directly impacted the transcription of the *IRF3* gene and IFN-β promoter-reporter activation (**Figures 8A, B**). We also found that VP1 potently inhibited SeV-triggered transcription of the *IRF3* and *IRF7* genes in HEK293T-*TPL2* ^-/-^ cells (**Figures 8C, D**). Similar to results shown in **Figure 6E**, reporter assays experiments indicated that endogenous TPL2 also inhibits VP1-mediated antagonistic effects (**Figure 8E**). As shown in **Figures 8F, G**, overexpression of TPL2 restored IFN-β signaling in VP1-overexpressing cells and in cells of the HEK293T-*TPL2^-/-^* cell line in a dose-dependent manner.

## Discussion

The exact mechanisms through which FMDV proteins interact with host cell proteins are not fully understood. Innate immunity is the first line of host defense against pathogenic infections. During their evolution, viruses have acquired numerous strategies to evade the host immune system. The FMDV L protease targets LGP2 helicase, which ultimately results in lower levels of IFN-β and antiviral activity (41). Furthermore, the downregulation of IKK/NF-κB has been observed in human lung cancer cells that express recombinant FMDV VP1 (42). In this study, we found that the host cellular TPL2 interacts with the FMDV structural protein VP1, thereby inhibiting virus replication. Overexpression of TPL2 resulted in decreasing FMDV replication; knockdown or knockout of TPL2 significantly enhanced FMDV replication *in vitro* (**Figure 1**). In order to confirm the role of TPL2 with respect to the host immune response and its ability to inhibit FMDV replication *in vivo*, the *Tpl2* gene-deletion led to a sharp increase in FMDV-associated mortality in infected suckling mice *in vivo* (**Figure 1**).

Transcription of interferon-stimulated genes encoding IFN-α, MX2 and CXCL10, IRF3, and IRF7 was significantly reduced in *Tpl2* gene-deleted suckling mice, while phosphorylation of IRF3 virtually disappeared (**Figure 2**). These data also confirmed both co-immunoprecipitation and co-localization of TPL2 with the virus VP1 protein (**Figure 3**). Moreover, it was further suggested that TPL2 could counteract VP1-mediated inhibition of the IFN-β signal pathway.

TPL2 has enzymatic activity and is a serine/threonine kinase in the MAPK signal transduction cascadeTPL2 has an important role in response to pathogens infection, including viruses, bacteria, and parasites (43). A previous study that delineated the signaling circuitry associated with virus sensing pathways suggested that TPL2 is a key regulator of the induction of inflammatory and antiviral genes in response to model viral ligands (44). Surprisingly, the role of TPL2 in innate immunity has been limited to models of bacterial and influenza virus infection; extensive studies of FMDV are lacking. Activation of transcription factors leads to the formation of an enhanceosome associated with the promoter of the primary type I interferon (IFN-I) gene product, IFN-β (45). Interestingly, our studies revealed that IKK and VISA promote type I IFN production when overexpressed in conjunction with TPL2 (**Figure 4**). A mutation of the TPL2 Thr290 site led to inhibition of the activation of IFN-I. Specifically, we found that when a plasmid expressing TPL2 is co-transfected with one that expresses IRF3, TPL2 can promote the activation of the IRF3/IFN-β signaling pathway. More studies are needed to understand the molecular mechanisms underlying TPL2-mediated IRF3/IFN-β activation and inhibition of the FMDV replication.

To explore the mechanisms through which TPL2 regulated virus-triggered expression of IFN-β, we first examined the molecular basis of TPL2 involvement in this process. Interestingly, our results revealed that TPL2 regulated IFN-β activation at the level of IRF3. We also demonstrated both co-immunoprecipitation and co-localization of host cellular TPL2 and IRF3 (**Figure 5**).

Phosphorylation of Ser62, Thr290, and Ser400 is necessary to induce TPL2 kinase activity (46–48). In this study, we constructed several plasmids that expressed deletion mutants of TPL2 and included key phosphorylation sites, including Thr290, Ser400, Asp270, and the TPL2 kinase active site Lys167. VP1-mediated impact at the IFN-β signaling pathway in the presence of TPL2 with mutations at Thr290 and Ser400 was not detected. Furthermore, the Thr290 mutation eliminated the TPL2-mediated IRF3 activation IFN-β signaling pathway. We also found that Thr290 of TPL2 is a key site for regulating the biological function of IRF3; VP1 can inhibit the activation of TPL2 at Thr290 (**Figure 6**). Our data revealed that VP1 could inhibit TPL2-mediated IRF3 activation and, thus, the IFN-β signaling pathway in a dose-dependent manner (**Figure 6C**). We further demonstrated that the VP1 (90-115) region significantly promoted significant inhibition of TPL2-mediated IFN-β signaling (**Figure 7**). Furthermore, our study uncovered that expression of VP1 inhibited the reporter activities of IFN-β promoters in a dose-dependent manner as TPL2 can significantly reduce VP1-mediated inhibition of the IFN-β signaling pathway (**Figure 8**).

**Figure 9:**
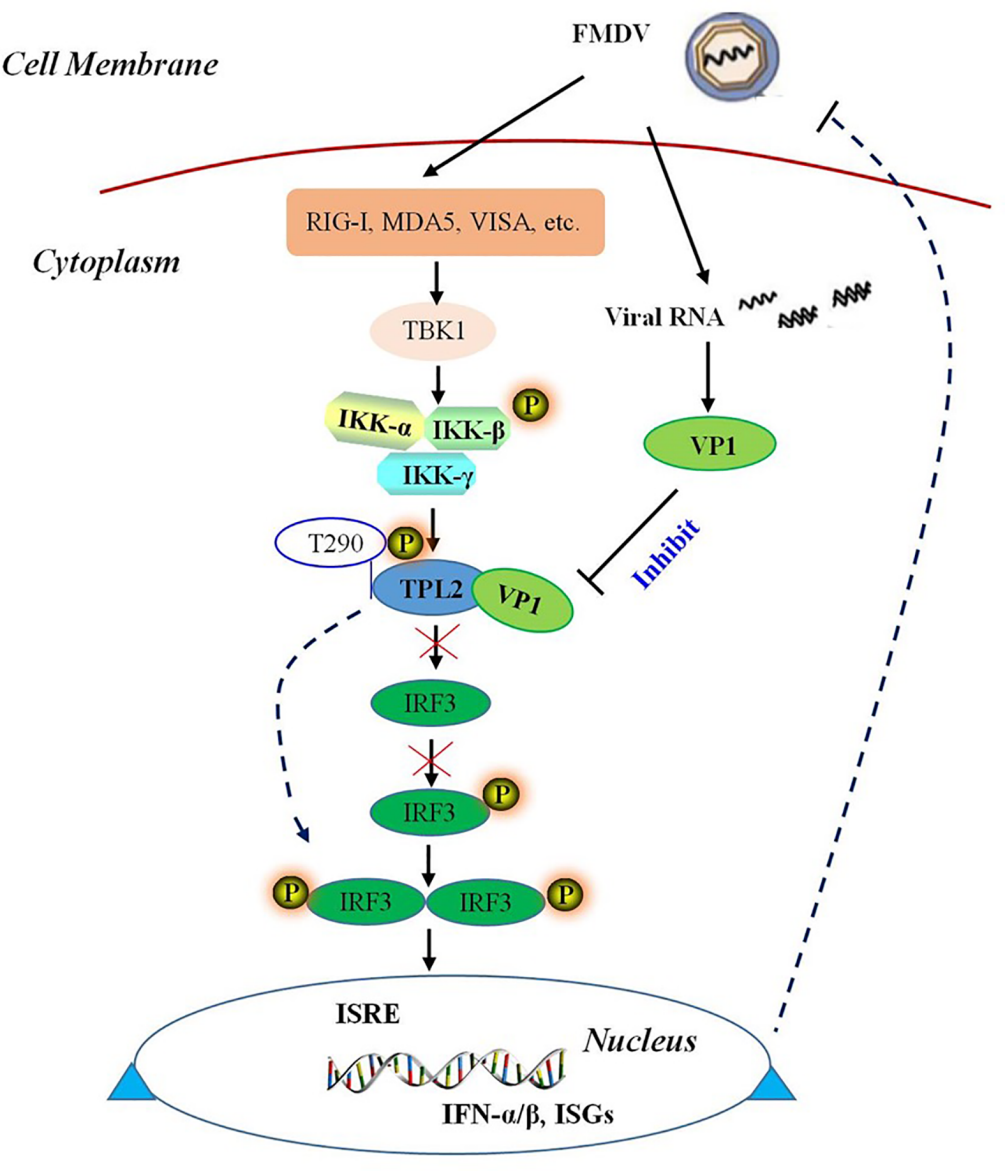
Schematic representation of a model VP1 inhibition of TPL2-mediated IRF3/IFN-β signaling. FMDV VP1 interacts with TPL2 and impairs its phosphorylation at Thr290. IFMDV capsid protein VP1 interacted with host TPL2 and inhibited TPL2-mediated IRF3/IFN-β signal pathway to immune escape and facilitate virus replication.

In conclusion, our study revealed that TPL2 is a novel binding partner of FMDV VP1. In addition, VP1 could inhibit the phosphorylation of TPL2 at Thr290. Our results also revealed that TPL2 Thr290 was the key functional site for promoting IRF3-mediated activation of the IRF3/IFN-β signaling pathway. As shown in **Figure 9**, FMDV capsid protein VP1 interacted with host TPL2 and inhibited the TPL2 site Thr290-mediated IRF3/IFN-β signal pathway for immune escape and to facilitate virus replication.

## Data Availability Statement

The datasets presented in this study can be found in online repositories. The names of the repository/repositories and accession number(s) can be found in the article/supplementary material.

## Ethics Statement

The animal study was reviewed and approved by “The Guide for Care and Use of Laboratory Animals” and in accordance with the national guidelines (License No. SYXK [GAN] 2014–003).

## Author Contributions

JuH and NW performed the majority of experiments and statistical analysis. CS carried out C57BL/6 infected FMDV experiments. MY, XZ, GX, DZ, JiH and WC helped with experiments. YJ, KZ, HZ and XL designed the study. JuH drafted the manuscript. All authors listed have made a substantial, direct, and intellectual contribution to the work and approved it for publication.

## Funding

This work was supported by the National Natural Science Foundation of China (NSFC-31972684) and National Science and Technology Support Plan (2015BAD12B04).

## Conflict of Interest

The authors declare that the research was conducted in the absence of any commercial or financial relationships that could be construed as a potential conflict of interest.

The reviewer LP declared a shared affiliation, with no collaboration, with the authors to the handling editor at the time of review.
