# Corrigendum: Foot-and-Mouth Disease Virus Capsid Protein VP1 Antagonizes TPL2-Mediated Activation of the IRF3/IFN-β Signaling Pathway to Facilitate the Virus Replication

**DOI:** 10.3389/fimmu.2021.686494

**Published:** 2021-08-06

**Authors:** Junhong Hao, Chaochao Shen, Nannan Wei, Minghao Yan, Xuegang Zhang, Guowei Xu, Dajun Zhang, Jing Hou, Weijun Cao, Ye Jin, Keshan Zhang, Haixue Zheng, Xiangtao Liu

**Affiliations:** State Key Laboratory of Veterinary Etiological Biology, National Foot-and-Mouth Disease Reference Laboratory, Lanzhou Veterinary Research Institute of Chinese Academy of Agriculture Science, Lanzhou, China

**Keywords:** foot-and-mouth disease virus, viral protein 1, tumor progression locus 2, interferon regulatory factor 3/interferon-β, immune escape

In the original article, there was a mistake in the legend for **Figure 6** as published. The legend of **Figure 6A** was not detailed enough and could easily be misunderstood. There was no description of the vector plasmid control. The correct legend appears below. The authors apologize for this error and state that this does not change the scientific conclusions of the article in any way. The original article has been updated.

In the original article, there was a mistake in **Figure 6** as published. We forgot to mark the two lanes of control plasmids (pCMV-3N-Myc) in the left Myc-TPL2 image in **Figure 6A** during the preparation and proof stage of our paper. This mistake led to the result that the actin control line included 12 lanes, while the Myc-TPL2 line contained 10 lanes in **Figure 6A**. There was no description of the transfected empty plasmids in the legend of **Figure 6A**. The corrected **Figure 6A** appears below.

The authors apologize for this error and state that this does not change the scientific conclusions of the article in any way. The original article has been updated.

**Figure 6:**
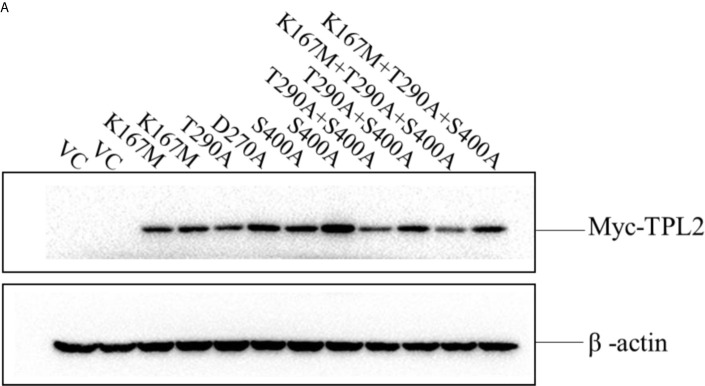
**(A)** HEK293T cells were transfected with TPL2 functional site mutant plasmids. Vector plasmids (pCMV-3N-Myc) were used as vector control (VC). Except for plasmids of T290A and D270A, the others occupied two lanes.

## Publisher’s Note

All claims expressed in this article are solely those of the authors and do not necessarily represent those of their affiliated organizations, or those of the publisher, the editors and the reviewers. Any product that may be evaluated in this article, or claim that may be made by its manufacturer, is not guaranteed or endorsed by the publisher.

